# Antimicrobial Capacity and Surface Alterations Using Photodynamic Therapy and Light Activated Disinfection on Polymer-Infiltrated Ceramic Material Contaminated with Periodontal Bacteria

**DOI:** 10.3390/ph13110350

**Published:** 2020-10-29

**Authors:** Elzahraa Eldwakhly, Selma Saadaldin, Alhanoof Aldegheishem, Marwa Salah Mostafa, Mai Soliman

**Affiliations:** 1 Department of Fixed Prosthodontics, Faculty of Dentistry, Cairo University, Cairo 12613, Egypt; zeldwakhly@yahoo.com; 2 Clinical Dental Science Department, College of Dentistry, Princess Nourah Bint Abdulrahman University, P.O. Box 84428, Riyadh 11564, Saudi Arabia; msmustafa@pnu.edu.sa; 3 Division of Prosthodontics, Schulich School of Medicine and Dentistry, Western University, London, ON N6A 3K7, Canada; selsaadaldin@gmail.com; 4 Department of Medical Microbiology and Immunology, Faculty of Medicine, Cairo University, Cairo 12613, Egypt; marwa75jan@yahoo.com

**Keywords:** photodynamic therapy, laser, ceramics, bacteria, decontamination

## Abstract

This study determined the antimicrobial efficiency of light-activated disinfection (LAD) and photodynamic therapy (PDT) on polymer-infiltrated ceramic network (PICN) material contaminated with three periodontal bacteria and explored if PDT and LAD cause PICN surface alterations. Sixty PICN discs were contaminated with *Tannerella forsythia*, *Porphyromonas gingivalis*, and *Treponema denticola* and randomly divided into five groups (*n* = 12 samples/each) according to the treatment groups: Group PDT—PDT (630 ± 10 nm diode laser) with methylene blue; Group DL—808 nm diode laser in contact mode without photosensitizer; Group MB–methylene blue without light application; Group CHX—0.12% chlorhexidine digluconate solution and; Group NT—no treatment. Each disc was then placed in tubes containing phosphate buffered saline (PBS) and vortexed for 30 s to remove the remaining bacteria from the discs. A total of 10× serial dilutions were performed followed by plating of 30 μL of suspension on Brucella agar plates. The colony forming units (CFU) were calculated after 72 h. PICN discs with the attached biofilms were used for confocal microscopy investigation for live/dead bacterial viability. A random single sample from each group was selected to study the bacterial adherence and topographical alterations on PICN discs under scanning electron microscope (SEM). The PDT group showed higher reduction for each bacterial species and total counts of bacteria assessed followed by the DL group (*p* < 0.05). When compared with MB group, the two laser groups were significantly superior (*p* < 0.05). The MB group did not show significant differences for any bacteria when compared to NT. The bacteria with the CHX group and DL groups appeared dead with few areas of surviving green stained bacteria. The PDT group showed the highest dead cell count (*p* < 0.05). PDT and DL groups indicate no significant changes on the surface compared to the sterile PICN discs on visual assessment. Photodynamic therapy produced superior periodontal bacteria reduction over the surface of PICN surface. PDT group showed higher reduction for each bacterial species and total counts of bacteria assessed followed by the DL group. Both PDT and DL treatment strategies are effective without producing surface alterations on PICN.

## 1. Introduction

Biological characteristics of indirect restorative materials is pertinent especially for fixed prostheses such as crowns and bridges. Such prostheses are in direct contact with the gingival tissue and can extend down to a certain depth inside the gingival crevice [1]. Hence, a connection establishes between the internal soft tissues with the external oral environment which creates an appropriate seal in order to form a hermetic barrier to protect periodontal soft and hard tissues from bacterial insult [2]. If bacterial penetration exceeds a limit, both hard and soft tissue destruction occurs in the form of bone resorption and soft tissue recession which consequently leads to the fixed prostheses failure and compromised esthetics [3,4].

Resin-based composites and ceramics are commonly used materials for dental restorations. However, these materials possess some limitations in terms of clinical performance either in terms of polymerization shrinkage, marginal adaptation, anatomic shape, or color match [5]. For optimizing the clinical performance of these materials, the manufacturers combined composite resins and ceramics to produce material by the name polymer-infiltrated ceramic networks (PICNs) [6,7]. Among these, polymer-infiltrated ceramic crowns (PICC) have gained more attention due to their superior mechanical and esthetic properties.

Like any other dental restorations, crowns in the oral cavity tend to create microbial plaque within the gingival crevice primarily due to improper oral hygiene, or other reasons such as inaccurate tooth preparation or ill-fitting prostheses [8]. Consequently, this leads to the development of specific oral infections called periodontal diseases, if untreated, may lead to crown failure and eventual tooth loss [8]. Periodontal inflammation is caused by the adhesion of anaerobic microorganisms such as *Porphyromonas gingivalis*, *Tannerella forsythia* and *Treponema denticola* [9]. Several therapeutic strategies are employed in order to eliminate the periodontal bacteria and reduce the severity of the infection. The most widely performed method is to disinfect the oral cavity by performing dental scaling and use of adjunctive chlorhexidine biguanide [10,11]. However, it is often difficult to eliminate the bacteria from inaccessible deep periodontal spaces between the crown and tooth junction [12]. Therefore, to surmount this limitation, other promising therapeutic strategies such as diode laser (DL) therapy and photodynamic therapy (PDT) have been introduced and being researched upon for more than three decades [13]. Light-activated disinfection is based on intensification of electromagnetic fields excited by light waves to emit well-collimated, coherent, and monochromatic laser beam [14]. On the other hand, PDT is a well-known technique which relies on laser application of specific wavelength and involves the excitation of photosensitizer dye molecule from ground singlet state to hyper triplet state in the presence of oxygen to form highly reactive singlet oxygen and other reactive oxygen species. These molecules are highly fatal and facilitates bacterial cell death [15].

To the authors knowledge, no study has been performed that assessed antimicrobial capacity against periodontal bacteria and evaluated surface alterations using PDT and DL. We aimed to evaluate the antimicrobial efficacy of PDT and DL on PICN discs contaminated with three periodontal species and investigate if the PDT and DL cause PICN surface alterations. The null hypotheses of the study were: (i) the laser therapies would not affect the microbial viability over the surface of the PICC and (ii) the laser therapies would not produce any surface alterations on the PICC.

## 2. Results

### 2.1. Antibacterial Testing

**Figure 1:**
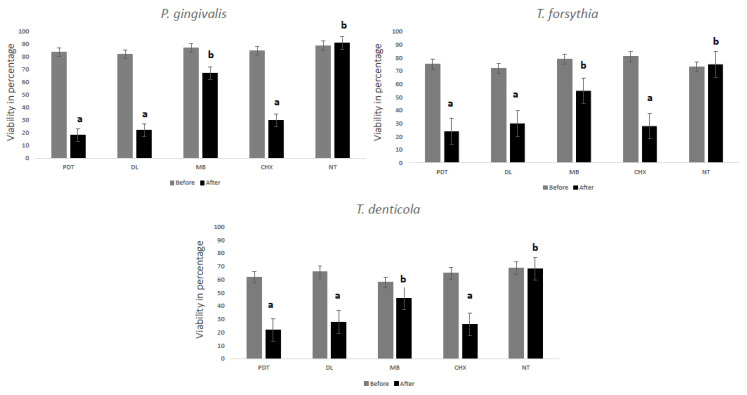
MTT assay showing relative percentage of *P. gingivalis*, *T. forsythia* and *T. denticola* biofilm viability with different treatment groups. Dissimilar letters indicate statistical significance between groups using ANOVA test followed by Tukey’s test.

Table 1 demonstrates the reduction of each bacterial species and the total bacterial count. Intergroup comparison showed statistically significant differences with regards to the three bacterial species individually and the total bacterial count (*p* < 0.05). For total colony forming units (CFUs), all the groups showed statistically significant reduction compared with the no treatment (NT) group (*p* < 0.05). It is noted that the reduction was >98% in each group. The PDT group showed the highest reduction for each bacterial species and total counts of bacteria assessed followed by the DL group (*p* < 0.05). When compared with MB group, the two laser groups were significantly superior (*p* < 0.05). The MB group did not show significant differences for any bacteria when compared to NT. Figure 1 shows microbial cell viability in percentage for all the bacteria assessed. It is noted that the lowest bacterial viability was seen in PDT, DL and CHX groups with no significant difference between the groups (*p* > 0.05).

### 2.2. Live/Dead Assay

**Figure 2:**
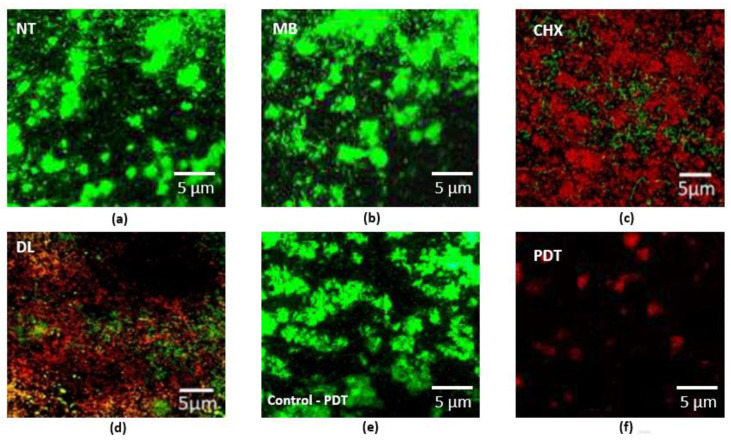
Representative CLSM images of periodontal bacteria grown on PICN discs are shown. (**a**) The control specimens with no treatment (NT) and (**b**) MB specimens are shown as green densely clustered colonies with almost no areas of dead cell; (**c**) represents data for CHX treated specimens. The bacteria in the CHX and (**d**) DL groups appeared dead with few areas of surviving green stained bacteria, indicating survivability; (**e**) Specimen from PDT group before decontamination (control for PDT) and (**f**) post decontamination indicating the highest dead cell count and destroyed bacteria that could be removed after treatment.

The CLSM images of bacteria cultured on PICN discs are shown in Figure 2. The dense green colonies (Figure 2a,b) with almost no area of dead bacterial cells represent the NT and MB specimens respectively. The bacteria with the CHX group (Figure 2c) and DL (Figure 2d) groups appeared dead with few areas of surviving green stained bacteria. The PDT group (Figure 2f) showed the highest dead cell count (*p* < 0.05). Figure 2e,f demonstrates pre-and post-treatment using PDT indicating the highest dead cell count and destroyed bacteria.

### 2.3. Surface Characterization

**Figure 3:**
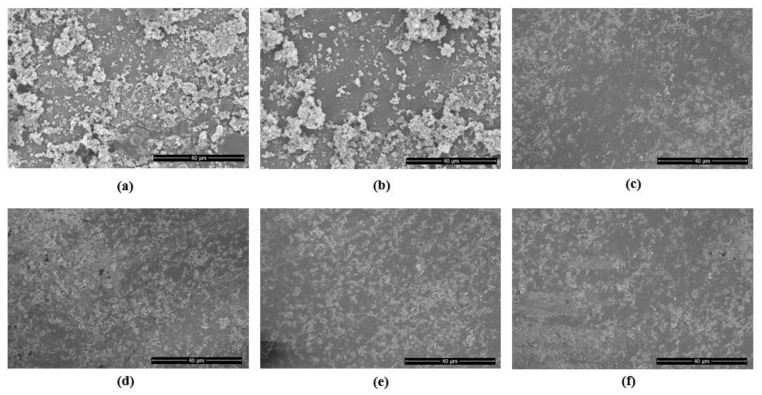
Representative SEM images of: (**a**) periodontal niche grown on PICN discs showing dense colonies (NT control specimen) (**b**) The specimen with MB shows no reduction in clustered colonies; (**c**) represents disc with CHX treatment; (**d**) DL therapy and; (**e**) PDT. The specimen (**f**) is shown to compare the sterile PICN disc (magnification 1500×).

SEM investigations revealed a thick periodontal niche grown over the surface of the PICN disc (NT group) (Figure 3a). The representative SEM images taken from treatment groups are shown in Figure 3b–e. 

DL (Figure 3d) and PDT (Figure 3e) groups indicate no significant surface alterations when compared with the image taken from the sterile PICN discs of the same type. On visual examination, they appeared to be the same as the surface of the sterile PICN disc (Figure 3e).

## 3. Discussion

The present study aimed to evaluate the antimicrobial efficacies of laser therapies (PDT and DL therapy) for surface decontamination of PICN and to examine if laser therapies could produce surface alterations over PICN surface. The outcomes of the present in-vitro study did not back our first null hypothesis and provide significant reduction of periodontal bacteria over the PICN surface. However, the second null hypothesis was backed in terms of laser therapies producing no surface alterations over PICN. This to our best knowledge, has never been investigated before and reports the first study in literature.

A plethora of basic and clinical research is being performed to test the efficacy of PDT in periodontics and implant dentistry [16,17,18,19,20]. It is proved to be a promising technique for combating periodontal and peri-implant infections around teeth and dental implants, respectively. With the growing number of dental crowns being fixed over teeth or dental implants, there is a rising need to investigate such new treatment methods for treating dental infections. It is well-known that in order to treat periodontal or peri-implant infections, it is imperative to reduce or eliminate the periodontal bacteria around the sulcus where crowns are fixed with the tooth or dental implant surface [21]. Photodynamic therapy or diode laser therapy offers maximum therapeutic outcome that is not produced by mechanical debridement or other chemicals alone such as the use of chlorhexidine [22,23]. This is true and based on the premise of how PDT works.

The mechanism of PDT is primarily based on the use of photosensitizer dye molecule that is taken up by the bacterial cell membrane that creates reactive oxygen species and other cytotoxic molecules in the presence of laser light which helps to deteriorate pathogens [15]. Our outcomes reported that PDT showed superior results as compared to other techniques reflecting how PDT could maximize the potential of antimicrobial therapy. It is important to further describe the proposed model that could be subjected to interactions by local microbiological environment [24]. The impact of PDT and LAD on various cells including microbiota, human cells and proinflammatory cytokine levels is well studied [25,26]. Future studies are warranted to test the impact of PDT and LAD on PICN on local periodontal environment including resident periodontal cells and microbiome.

The outcomes of our study represented differences among study groups and all experimental groups showed periodontal bacterial reduction compared to the group that did not undergo any treatment. In several studies performed on other materials such as titanium or zirconia surfaces, using PDT or diode laser therapy was shown to be effective in reducing bacteria [27,28,29]; however, the decreases were lesser than the reductions obtained in our study. Such differences may be attributed to the type of ceramic material surface being studied and according to the previous published literature, the adherence of bacteria to attach to PICN and zirconia is significantly lower as that of bacteria attached to titanium surface which is due to the significant difference in the surface free energy and surface roughness [30,31,32]. Therefore, we hypothesize that the periodontal bacteria did not adhered well enough on the PICN surface after 72 h of incubation period along with the rinsing of photosensitizer MB that may have caused additional detachment of the periodontal niche form PICN surface.

The present antibacterial results of PDT over PICN surface are in accordance with the previous studies conducted on titanium implant surfaces. For instance, Azizi et al. in their in-vitro study reported PDT and light activated disinfection showed high effectiveness against oral bacteria on zirconia implant surfaces [29]. Similarly, Sayar et al. [33] also reported significant reduction of a pathogenic periodontal bacteria *Aggregatibacter actinomycetemcomitans* over the titanium discs using PDT. In addition, other pre-clinical studies have investigated the impact of decontamination over other types of dental restorations [34]. For this purpose, the physical properties of dental restorations highlight important role of the impact of phototherapy against microbial niche on several types of dental biomaterials [35,36]. Furthermore, previous studies have used different laser parameters, such as different types of photosensitizers with different laser wavelengths. While these studies have indicated that PDT demonstrated the highest level of antibacterial efficacy over different types of material surfaces [37]. In relation to the laser parameters, whether PDT or DL have significant impact on surface alterations on dental restorations is still unknown [37].

Some limitation exists in the present in vitro study. The present study did not assess the cell viability and how human gingival fibroblast cells interact with the PICN material with the potential application of PDT. The oral cavity and their related tissues are rich in stem cells that are conveniently harvestable. These resident stem cells also act as anti-inflammatory and immunomodulatory elements in the local biological niche [38,39]. Such interactions with PICN material and the effects PDT and DL warrants investigation. Another important limitation exists regarding the use of conventional in vitro microbial growth/culture. Future studies should rather focus on safe and predictable in vitro culture protocols especially with those working without any additive on microbial cells and soft tissues, for instance, Bovine Serum Albumin (BSA) or Fetal Bovine Serum (FBS) to apply safely on human cells [40]. In addition, the combination of a single photosensitizer with diode laser was investigated. The including of different photosensitizer with different laser type may provide a robust comparison between laser and other non-laser groups.

With the outcomes of our study showing significant antibacterial efficacy with PDT and DL, these therapeutic strategies could be translated into clinical applications. However, the cost of the laser treatment, handling of the instrument, training/expertise with appropriate guidance with potential side effects should be taken into consideration.

## 4. Materials and Methods

### 4.1. Study Ethics and Samples

The study protocol was approved by the Faculty of Dentistry of Princess Nourah Bint Abdulrahman University under the protocol identification number: PNU-76-0001. Power analysis was performed prior to the study by keeping the alpha value at 5% and study power of 95% and 5 investigated groups [27,41]. For these incorporated parameters, it was necessary to have 12 samples/discs per group. For this study, 60 PICN discs (Vita Enamic, Vita Zahnfabrik, Bad Säckingen, Germany) were sectioned with the dimensions 10 × 10 × 3.0 mm using slow speed isomet (Isomet, Buehler, Evanston, IL, USA) under cooling water. The sectioned PICN discs were thoroughly rinsed with distilled water and further sonicated in 99% isopropanol placed in ultrasonic bath for 5 min.

### 4.2. Microbial Contamination of the PICN Discs

Bacterial contamination of the PICN follows the steps described in the previous study [29]. Suspensions from the three periodontal microbes [*P. gingivalis* (ATCC33277), *T. forsythia* (ATCC35405) and *T. denticola* (ATCC43037)] were prepared. All bacteria were grown separately on blood agar plates except for *P. gingivalis* which were grown on blood agar supplemented with tryptic soy broth. All bacteria were grown under anaerobic conditions. The bacterial suspension was mixed using thioglycolate in a joint suspension. A 600 nm (equivalent of 0.5 McFarland standard containing 1 × 10^8^ CFU/mL) density was adjusted in a densitometer (Shimadzu CS 920, Tokyo, Japan). All PICN discs were placed in their full length in 2 mL Eppendorf tube (Hamburg, Germany) containing 250 µL of the bacterial suspension and incubated anaerobically for 72 h in BD GasPak™ jar (BBl, Becton Dickinson and Company, Sparks, MD, USA).

### 4.3. Therapies

After 72 h of incubation period in the anaerobic jars, the PICN discs were randomly divided into five groups (12 discs/group).

#### 4.3.1. Photodynamic Therapy Group (PDT)

The contaminated PICN discs were sprayed with 150 µL of MB (Sigma Aldrich, St. Louis, MO, USA) and left undisturbed for 60 s. The discs were washed thoroughly with PBS solution and later irradiated with 630 ± 10 nm diode laser (FotoSan, CMS Dental APS, Copenhagen, Denmark) with power output 100 mW and density of 2000–4000 mW/cm^2^ for 60 s. The power fluence was set at 90 J/cm^2^. The distance between the diode laser tip and the specimen was maintained at 1 mm and spot area of 0.502 cm^2^.

#### 4.3.2. Diode Laser Group (DL)

In this group, the contaminated PICN were treated using an 808 nm diode laser (Lasercat 500, Medsolution, Radolfzell, Germany) in contact mode. The laser tip was positioned at 1 mm and sequentially, until the irradiation time reached 1 min per surface of the PICN section.

#### 4.3.3. Methylene Blue Group (MB)

The PICN discs in this group were immersed in 3 mL of MB dye solution (Sigma Aldrich) of concentration 1 mg/mL for 60 s. Later on, the discs were taken out and rinsed thoroughly with PBS solution.

#### 4.3.4. Chlorhexidine Group (CHX)

The bacterial contaminated PICN discs were immersed in 3 mL of 0.12% chlorhexidine (CHX) digluconate solution (Sigma Aldrich) and left for 2 min. The treated specimens were later thoroughly irrigated with PBS solution to remove the excess CHX solution.

#### 4.3.5. No Treatment (NT)

The fifth group served as control specimens in which none of the decontamination technique was performed and left untreated.

### 4.4. Microbial Analysis

Immediately after performing therapies, each PICN discs were immersed in 600 µL PBS solution placed in 2 mL Eppendorf tube. The samples were vortexed for 30 s to detach the bacteria from the specimen surface. 100 µL from each Eppendorf tube were transferred to Mueller Hinton broth (100 µL). Subsequently, 20 μL of PBS from each Eppendorf tube was transferred to a microplate well containing 180 μL of Mueller Hinton Broth. A total of 10-fold serial dilutions were performed in 96-well plates followed by the inoculation of 30 μL of the suspension from each well and plated to Brucella agar plates. Incubation of the plates was performed for 72 h in anaerobic conditions, and CFU were counted later. Distinctive colonies were detected using MALDI Biotyper (Bruker Daltonics, Leipzig, Germany) with visual analysis.

### 4.5. Confocal Laser Microscopy

PICN discs with the attached biofilms were used for confocal microscopy investigation for Live/Dead Bacterial Viability. The viability of bacteria was checked using a confocal laser scanning microscope (CLSM; Fluoview FV 1000, Olympus, Tokyo, Japan). LIVE/DEAD BacLight stain (Invitrogen, Carlsbad, CA, USA) was used after mixing according to the manufacturer’s instructions. The PICN specimens were incubated for 30 min in the dark, excessive stain removed and analyzed with CLSM using light emission between 500 and 550 nm with an excitation wavelength of 488 nm and ×100 objective.

### 4.6. Scanning Electron Microscopy

A random single sample from each group was selected to study the bacterial adherence and topographical alterations on PICN discs under scanning electron microscope (Tescan VEGA3, Tokyo, Japan). The specimens were fixed using 4% paraformaldehyde and 2% glutaraldehyde in 0.1 M sodium cacodylate buffer (NaCac) with pH 7.4 and stored overnight. Subsequently, the samples were treated with 2% osmium tetraoxide and dehydrated in a series of ethanol concentrations (60–100%). The samples were later processed for critical point drying using CO_2_. The dried PICN discs were taped in double sided copper tape and sputter coated with 5 nm platinum coating. The PICN discs were imaged at 10 kV using through-lens detector (TLD) for secondary electron imaging. Representative images from each group were selected for depiction.

### 4.7. Statistical Analysis

Statistical data were analyzed using statistical package of SPSS (v22 IBM Corp., Armonk, NY, USA). Normality testing was performed using Kolmogorov-Smirnov test. Alpha level was set at <0.05. The differences between the groups for each periodontal bacterium assessed and overall count of bacteria were compared using ANOVA test. Multiple comparisons were applied using Tukey-Kramer method. The data for bacteria were log transformed using the following formula [29]:

L = log_10_ (N + 1) (1)

Bacterial reduction and their percentages compared to the NT group was computed using the following formula [29]:

1 − T/C = 100 × (1 − T/C)%
(2)

where T = mean value for each tested group and C = NT group.

## 5. Conclusions

Photodynamic therapy produced superior periodontal bacterial reduction over the surface of PICN. PDT group showed higher reduction for each bacterial species and total counts of bacteria assessed followed by the DL group. Both PDT and DL treatment strategies are effective without producing surface alterations on PICN.

## Figures and Tables

**Table 1:** Mean and standard deviation of periodontal bacteria assessed with total counts in logarithms.

Groups	*P. gingivalis*			*T. forsythia*			*T. denticola*			Total		
	Mean	SD	*p*-Value	Mean	SD	*p*-Value	Mean	SD	*p*-Value	Mean	SD	*p*-Value
PDT	0.6 ^a^	1.1	<0.001 *	0.5 ^a^	0.7	<0.001 *	0.5 ^a^	0.8	<0.001 *	0.7 ^a^	0.9	<0.001 *
DL	0.8 ^a^	1.2		0.8 ^a^	0.9		0.6 ^a^	0.7		0.9 ^a^	1.0	
MB	5.7 ^b^	1.0		5.6 ^b^	1.3		5.4 ^b^	1.1		6.8 ^b^	1.3	
CHX	1.0 ^a^	1.1		0.9 ^a^	1.0		0.8 ^a^	0.9		1.3 ^a^	1.4	
NT	6.5 ^b^	1.3		6.2 ^b^	0.9		6.1 ^b^	1.3		7.1 ^b^	0.9	

Dissimilar letters indicate statistical significance between groups; * *p*-value obtained using ANOVA test.
